# Polygenic risk score analyses of symptoms and treatment response in an antipsychotic-naive first episode of psychosis cohort

**DOI:** 10.1038/s41398-018-0230-7

**Published:** 2018-08-31

**Authors:** Marcos Leite Santoro, Vanessa Ota, Simone de Jong, Cristiano Noto, Leticia M. Spindola, Fernanda Talarico, Eduardo Gouvea, Sang Hyuck Lee, Patricia Moretti, Charles Curtis, Hamel Patel, Stephen Newhouse, Carolina Muniz Carvalho, Ary Gadelha, Quirino Cordeiro, Rodrigo Affonseca Bressan, Sintia Iole Belangero, Gerome Breen

**Affiliations:** 1 Genetics Division, Federal University of São Paulo (UNIFESP), São Paulo, SP Brazil; 2 Interdisciplinary Laboratory of Clinical Neurosciences, Federal University of São Paulo (UNIFESP), São Paulo, SP Brazil; 3 Social, Genetics & Developmental Psychiatry Centre (SGDP), Institute of Psychiatry, Psychology and Neuroscience at King’s College London, London, UK; 4 Department of Psychiatry, Federal University of São Paulo (UNIFESP), São Paulo, SP Brazil; 5 Centro de Atendimento Integrado em Saúde Mental (CAISM), Santa Casa de Misericórdia de São Paulo, São Paulo, SP Brazil; 6 NIHR Maudsley Biomedical Research Centre, South London and Maudsley NHS Trust & King’s College London, London, SE5 8AF UK

## Abstract

In this study, we aimed to test if the schizophrenia (SCZ) polygenic risk score (PRS) was associated with clinical symptoms in (a) the first episode of psychosis pre-treatment (FEP), (b) at nine weeks after initiation of risperidone treatment (FEP-9W) and (c) with the response to risperidone. We performed a detailed clinical assessment of 60 FEP patients who were antipsychotic-naive and, again, after nine weeks of standardized treatment with risperidone. After blood collection and DNA isolation, the samples were genotyped using the Illumina PsychArrayChip and then imputed. To calculate PRS, we used the latest available GWAS summary statistics from the Psychiatric Genomics Consortium wave-2 SCZ group as a training set. We used Poisson regression to test association between PRS and clinical measurements correcting for the four principal components (genotyping). We considered a p-value < 0.0014 (Bonferroni correction) as significant. First, we verified that the schizophrenia PRS was also able to distinguish cases from controls in this south-eastern Brazilian sample, with a similar variance explained to that seen in Northern European populations. In addition, within-cases analyses, we found that PRS is significantly correlated with baseline (pre-treatment) symptoms, as measured by lower clinical global assessment of functioning (−GAF), higher depressive symptoms and higher scores on a derived excitement factor. After standardized treatment for nine weeks, the correlation with GAF and the excitement factor disappeared while depressive symptoms became negatively associated with PRS. We conclude that drug (and other treatments) may confound attempts to understand the aetiological influence on symptomatology of polygenic risk scores. These results highlight the importance of studying schizophrenia, and other disorders, pre-treatment to understand the relationship between polygenic risk and phenotypic features.

## Introduction

Schizophrenia (SCZ) is a severe mental disorder affecting ~1% of the population and is characterized by the presence of psychosis and other features, such as negative (i.e., flattened affect and social withdrawal) and disorganization symptoms (e.g., disorganized speech and behaviour). Symptomatic and psychosocial deterioration progress rapidly during the period just after the onset of the disorder, termed the first episode of psychosis (FEP)^1^. Moreover, reports indicate that brain abnormalities and cognitive deficits are already present in the FEP^1^, even though patients are not affected yet long exposure to antipsychotics^2,3^.

SCZ is highly heritable (~80%)^4^. The most recent Genome-Wide Association Study (GWAS) for SCZ in the Psychiatric Genomics Consortium (PGC) wave 2 (PGC2) tested the association of millions of single-nucleotide polymorphisms (SNPs) and other types of genetic variations in ~34,000 cases and ~113,000 controls, and was particularly successful in uncovering new genes and pathways for the disorder^5^. For SCZ (and other psychiatric disorders), it is now well accepted that, while no single variant accounts for a large proportion of cases, thousands of genetic variants act together to confer the majority of the genetic risk for the disorder—a polygenic architecture of risk^6,7^.

Purcell et al.^8^ developed and applied the method proposed by Wray et al.^7^ to calculate a polygenic risk score (PRS) explaining 2–3% of variance in SCZ case-control status^7,8^. With the large increases in sample size enabled by international GWAS consortia, the SCZ PRS has become more powerful. Using the PGC2 SCZ GWAS as a training sample^5^, predictive SNPs achieving a nominal *p*-value threshold can be selected and the PRS of an individual in independent sample can be calculated. More specifically, the effect size estimated in the training sample for each SNP’s risk allele is multiplied by the number of risk alleles present in an individual. This is then summed across all variants selected in the genome to yield a PRS for every individual in a training sample^8,9^.

Such a PRS has a much larger effect size than any single genetic variant and does not need a large sample size for the target sample as long as it is estimated from a very large training sample^9^. The SCZ PRS represents a genetic estimate of liability to the disorder and is a normally distributed quantitative trait that can be applied in many ways. For SCZ, the PRS has been correlated with quantitative variables, such as severity of symptoms^10^ and prefrontal activity^11^. For bipolar disorder, PRS has been correlated to function and brain structures in individuals at risk^12,13^, and, for depression, it has been correlated with the reduction of the cortical volume in specific regions^14^. A recent paper by Vassos et al.^15^ reported that SCZ PRS is associated with diagnosis inFEP patients. One recent study reported a positive correlation between SCZ PRS and negative symptoms in an (unaffected) adolescent population cohort^16^. Recently, another article found positive associations between a genetic (rather than polygenic) risk score generated with 84 SNPs with positive and negative symptoms at the FEP, but not after treatment^17^.

No study, to our knowledge, has examined the correlation of PRS with the untreated symptom profile of SCZ patients or the response to treatment in FEP patients. In this study, we test if the SCZ PRS is correlated with symptomatology, severity and response to antipsychotics during FEP in a serial longitudinal sample of initially treatment naive patients.

## Methods

### Recruitment and consent

We enrolled patients with FEP at admission to the Centro de Atenção Integral a Saúde Mental (CAISM), São Paulo. The study protocol was designed to address the acute but temporary lack of capacity in FEP patients at admission. When a patient was admitted meeting the inclusion criteria (below), medical staff explained the study to family members, provided printed information sheets and, if agreeing, families then signed a written informed consultee consent with the assent of the patient. At the follow-up assessment, the patients were directly consented into the study, provided they had capacity. If subjects lacked capacity at the follow-up assessment, consent was taken at a later stage when capacity was regained. The local Research Ethics Committee of Universidade Federal de São Paulo (CEP-UNIFESP 0603/10) and the national Brazilian Ethics Committee (CONEP-CAAE 33148114.6.0000.5505, CAAE 48242015.9.0000.5505) approved the research protocol.

### Longitudinal cohort of FEP patients

Our cohort of antipsychotic-naiveFEP patients includes 154 subjects recruited from a psychiatric emergency unit in São Paulo (Brazil). The diagnosis of a psychotic disorder was established by trained psychiatrists using Diagnostic and Statistical Manual of Mental Disorders, Fourth Edition (DSM-IV) criteria, using the Structured Clinical Interview for DSM-IV (SCID-I). Inclusion criteria were aged between 16 and 40 years without previous history of antipsychotic medication and with confirmed non-affective psychosis (SCZ, schizophreniform disorder or brief psychosis disorder diagnosis) after two months of treatment. Prior or current treatment with benzodiazepines was allowed. Patients with psychotic episodes due to a general medical condition, substance-induced psychotic disorder, intellectual disability, major depressive disorder or bipolar disorder were excluded.

A total of 60 patients met criteria for antipsychotic-naive FEP after the follow-up (FEP, *N* = 60). These patients were assessed at baseline and followed up for 9.03 ± 2.76 weeks of risperidone treatment. Four patients were taking benzodiazepines and one clonazepam, at baseline. During follow-up, besides risperidone, 12 were taking clonazepam and 7 mood stabilizers.

The healthy control group (*N* *=* 60) comprised age-gender-and-ethnicity-matched volunteers with no first-degree family history of psychotic disorders, who were evaluated by trained psychiatrists using a modified SCID-I to ensure no current or previous psychiatric diagnoses. Peripheral blood samples were collected in EDTA tubes at baseline and follow-up for patients and after psychiatric interview for controls.

### Clinical assessments

All psychiatrists had the same training at the “*Programa de Esquizofrenia da UNIFESP*” and the FEP patients were always assessed by the same psychiatrist at both time points for the following scales: (a) PANSS (Positive and Negative Syndrome Scale), (b) CGI (Clinical Global Impression Scale)^18^, (c) GAF (Global Assessment of Functioning Scale), (d) CDSS (Calgary Depression Scale for SCZ)^19^.

Symptom clusters (negative, positive, disorganization, excited and anxiety/depression) from the PANSS items^20^ were calculated using the algorithm from a previous study in a Brazilian population^21^. For more information about each symptom cluster, see Supplementary Table S1. Response to treatment was defined as a > 50% reduction in baseline PANSS total score^22^. GAF is the only scale where higher values represent less impairment; thus we transformed to them to negative values (referred to as −GAF).

### DNA isolation

Whole blood was collected into EDTA tubes, and genomic DNA isolation was performed using the Gentra Puregene Kit (Qiagen) according to the manufacturer’s protocol.

### Genomic arrays

The genotyping was performed at King’s College London using the Infinium PsychArray-24 BeadChip (Illumina) with a GWAS core backbone (~590 K markers) and specific content from the Psychiatric Genomics Consortium: https://www.med.unc.edu/pgc/psychchip.

### Quality control and imputation

For the quality control (QC) parameters, we removed SNPs with a minor allele frequency (MAF) < 1%, *Locus* missingness > 10% or Hardy–Weinberg disequilibrium significance < 0.00001. We also excluded individuals with missingness > 10% and an estimation of identity-by-descent > 0.12. Genotype imputation was performed using the https://imputation.sanger.ac.uk using as Reference Panel the Haplotype Reference Consortium (release 1) with 32,488 samples (39 M sites) and the Pre-phasing algorithm SHAPEIT2. After post-imputation QC, using the same parameters as above, ~ 9 M SNPs were analysed.

### Polygenic risk scores

For more information about how the scores are calculated, please see the Supplementary Material of Purcell et al.^8^. To generate the PRS we used the PRSice software (www.PRSice.info) default options. The SCZ sample from PGC2 (downloaded from https://www.med.unc.edu/pgc) was used as the training sample and our imputed genotyping sample as the target. The PGC2 SCZ PRS is generated from many individual samples that may represent more chronic and severe SCZ, such as patients on clozapine. This means the PGC PRS represents a powerful tool to understand the influence of SCZ risk on clinically important symptom dimensions pre-treatment. We performed *P*-value-informed clumping with a cutoff of *r*^2^ = 0.1 using a 250-kb window and calculated scores per individual for multiple *p*-threshold (ranging from 0.0001 to 0.5 with increments of 0.00005) including or excluding the MHC (major histocompatibility complex) region on chromosome 6, which has a complex linkage disequilibrium structure. Given that our sample is sampled from an admixed south eastern Brazilian population, we carefully assessed population stratification and used the first four components generated by plink1.9 software were used as covariables. Posteriorly, PRSice runs a regression to find the best *p*-threshold based on the explained variance (Nagelkerke’s pseudo-*r*^2^ correlation) and in our case gave PRSs based on the most FEP case-control variance explained.

### Statistical analysis

We used R for all statistical analysis. With the PRSs calculated for the case-control comparison, we used a generalized linear model to test PRS associations assuming a Poisson distribution (Poisson regression), which is more suitable for ordinal variables (such as psychiatric scales), using clinical traits as the dependent variable and the best *p*-threshold PRS with the first four principal components as the independent variables and covariates. As clinical outcome variables, we considered, for both time points, GAF score, total CGI score, total PANSS scores and the five PANSS dimension clusters suggested by Wallwork et al.^20^ and validated by Higuchi et al.^21^ in the Brazilian population. GAF values were transformed to negative values (−GAF), so all clinical variables were easily compared, with high values meaning high symptomatology. We defined as outliers those observations lying beyond 1.5 times the ‘Inter Quartile Range’ - the difference between 75th and 25th quartiles.

We applied the Bonferroni correction for multiple comparisons (number of psychiatric scales tested *N* = 36), considering as significant a *p*-value < 0.0014 (0.05/36). As the Brazilian population is known to be a highly admixture population, we first plotted case and controls principal components to check if they have similar background and then we did a sensitivity analysis considering only full European ancestry cases.

Using the residuals from the PRS with principal components, we tested if the available demographics could be potential confounders. Further, we tested if response to risperidone overall or within subtypes of FEP included in our study (SCZ or schizophreniform) was associated with SCZ PRS. First, we tested the change in symptoms from baseline to the follow-up and if the subtype of FEP was associated with the PRS using a Poisson regression. Second, we tested the correlation between the change in total PANSS and PRS using a Pearson correlation. Finally, we verified if there was an association of clonazepam or mood stabilizers with CDSS, CGI, GAF and PANSS symptoms that could be affecting the results.

## Results

Table 1 shows the clinical and demographic characteristics of the participants. Smoking rates significantly higher in cases than in controls. Patients showed improvement after nine weeks of risperidone treatment for all scales and symptom clusters, except for PANSS negative. Figure S8 shows a heatmap of the correlations among the tested clinical variables, demonstrating, a high correlation between PANSS depressive factor and CDSS, and between GAF and most clinical variables. Table S2 and S3 shows that there are no associations between demographics with either PRS or clinical variables.

**Table 1:** Clinical and demographic characteristics of the participants in this study

Variable	Healthy controls (*N*=59)	Antipsychotic-naive FEP (*N*=60)	FEP after treatment (*N*=60)	*p*-value
Gender (%)	M:34 (57.6%)	M:40 (66.7%)		0.309
Age in years; mean (SD)	25.97 (7.48)	25.63 (7.46)		0.808
Currently smoking (%)	*N* = 2 (3.6%)	*N* = 12 (23.5%)		0.002
Family history of psychosis (%)		*N* = 23 (50%)		
Cannabis use (%)		*N* = 18 (52.9%)		
Other drugs use (%)*		*N* = 13 (41.9%)		
Family income in US$/month (SD)		870.59 (792.31)		
BMI in kg/m^2^		23.58 (3.78)		
PANSS negative; mean (SD)		27.37 (10.51)	25.02 (9.28)	0.127
PANSS disorganization/cognition; mean (SD)		26.96 (8.58)	19.91 (6.22)	5.029 × 10^-8^
PANSS excitement; mean (SD)		24.69 (9.09)	13.22 (5.62)	7.84 × 10^-14^
PANSS positive; mean (SD)		34.75 (7.32)	21.23 (9.52)	2.46 × 10^-13^
PANSS depression/anxiety; mean (SD)		24.24 (8.79)	18.11 (7.85)	2.97 × 10^-5^
PANSS total		94.55 (20.94)	68.21 (20.31)	1.71 × 10^-10^
GAF; mean (SD)		31.21 (10.52)	55.47 (16.61)	1.34 × 10^-11^
CGI; mean (SD)		4.83 (0.72)	3.35 (1.26)	1.77 × 10^−11^
CDSS; mean (SD)		4.64 (5.04)	2.48 (4.27)	0.007

*M* male, *SD* standard deviation, *FEP* first-episode psychosis, *PANSS* Positive and Negative Syndrome Scale, *CGI* Clinical Global Impression Scale, *GAF* Global Assessment of Functioning Scale, *CDSS* Calgary Depression Scale for Schizophrenia

*Drugs including cocaine, sedatives, stimulants, hallucinogens, opioids and gases

### Brazilian admixture sample

Cases and controls showed similar principal components structure, and when analysing only the European ancestry individuals (self-declared and consistent with the genetic estimates) the direction and magnitude of associations remained the same (Table S5 and Figures S1, S7).

### Polygenic risk

**Fig. 1:**
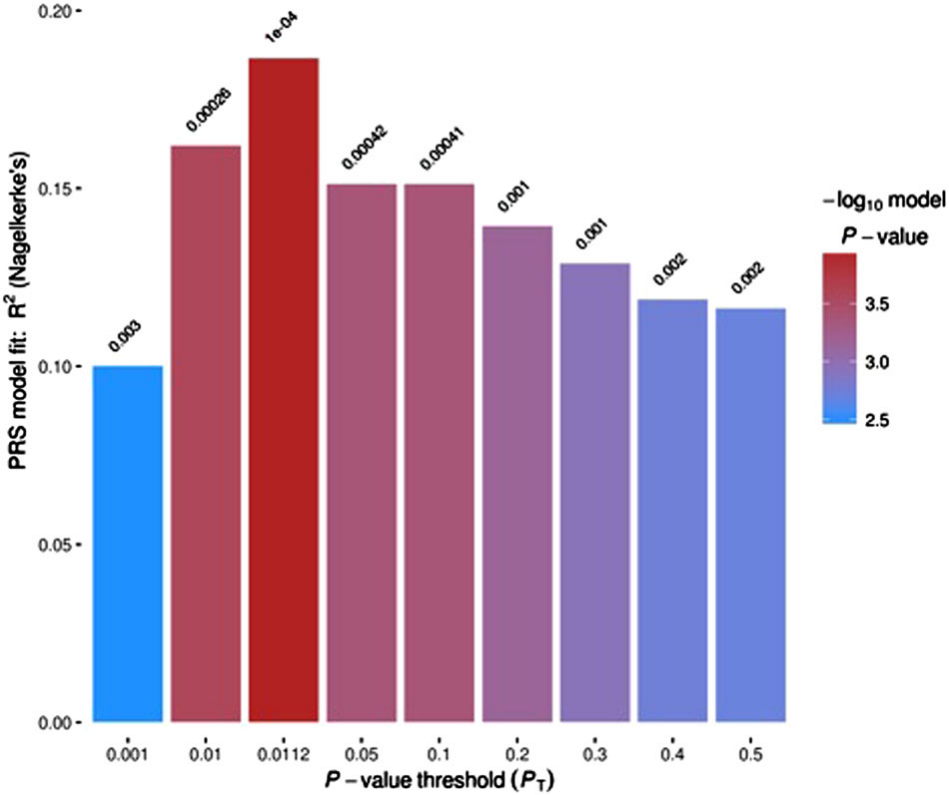
Graph from PRSice showing the explained variance (*y*-axis) for each *p*-threshold (*x*-axis) to identify cases and controls for our sample

The number of independent SNPs analysed for each threshold and cohort is described in Supplementary Table S4. With or without the MHC region, results were similar; thus, we carried on including the MHC region to increase the number of analysed SNPs and, consequently, the power of our analysis. The PRS was significantly different between cases and controls (Fig. 1) with a best *p*-threshold of 0.0112 (*N*_SNPs_ = 21,622) and an explained variance of 0.19 (Nagelkerk’s pseudo-*r*^2^). Figure S7 shows the normal distribution of the PRS for cases and controls, and Figure S10 shows the odds ratio (OR) of psychosis for quantiles of PRS.

### PRS and clinical variables relation

At baseline (antipsychotic naive FEP), we find a positive association PRS with the PANSS excitement factor (five-factor model) (*B* = 566.7; *p*-value = 0.0003) and a trend for association with –GAF (*B* = 436.1; *p*-value = 0.003). PRS showed a positively trend for depressive symptoms at baseline (CDSS total: *B* = 1042.3; *p*-value = 0.0039) but became significantly negatively associated with depressive symptoms after risperidone treatment (CDSS total: *B* = −1800.2; *p*-value = 0.0004). The results are summarized in Table 2 and Figures S2–S4.

**Table 2:** PRS correlation with clinical variable during the baseline and the follow-up

Time	Clinical variable		*N*	B	*p*-value
Baseline (antipsychotic naive FEP)	CGI		50	72.8	0.8436
	^a^−GAF		48	436.1	0.0030
	^a^CDSS total		51	1042.3	0.0039
	PANSS total		53	38.1	0.6390
	^a^PANSS positive		53	400.0	0.0278
	PANSS negative		53	−205.6	0.3048
	PANSS general psychopathology		53	−20.2	0.8886
	Five-factor model^21^	PANSS negative	53	−168.2	0.2655
		PANSS disorganization/cognition	53	−32.9	0.8281
		^b^PANSS excitement	53	566.7	0.0003
		PANSS positive	53	27.1	0.8382
		PANSS depression/anxiety	53	−112.8	0.4761
Follow-up (9 weeks treated with risperidone)	CGI		51	−137.4	0.7588
	−GAF		53	−132.8	0.2281
	^b^CDSS total		53	−1800.2	0.0004
	PANSS total		54	−113.4	0.2215
	PANSS positive		56	277.3	0.3141
	PANSS negative		56	−358.3	0.0894
	PANSS general psychopathology		54	−287.9	0.1140
	Five-factor model^21^	PANSS negative	56	−180.6	0.2329
		PANSS disorganization/cognition	56	−75.8	0.6608
		PANSS excitement	56	216.4	0.3048
		PANSS positive	56	10.0	0.9522
		^b^PANSS depression/anxiety	55	−575.0	0.0013

*PANSS* Positive and Negative Syndrome Scale, *CGI* Clinical Global Impression Scale, *GAF* Global Assessment of Functioning Scale, *CDSS* Calgary Depression Scale for Schizophrenia

^a^Significant *p*-values without Bonferroni correction

^b^Significant *p*-values with Bonferroni correction

Looking at response to risperidone, we analysed the five PANSS factors and other scales, we observed a positive association for ΔCDSS (*B* = 717; *p*-value = 0.0006) (Table 3). However, considering the total PANSS, there was no correlation between Δtotal PANSS and PRS (*t* = 0;62633, df = 49; *p*-value = 0.534; *r* = 0.089). Although a borderline association was observed between PANSS-excitement at the follow-up and mood stabilizer use (*N* = 7), the relationship between PRS and PANSS-excitement remained not significant even adding this as a covariate (*B* = −110, *p* = 0.94).

**Table 3:** PRS association with clinical variables of risperidone treatment response

Clinical variables (follow-up - baseline)		*N*	B	*p*-value
Five-factor model^21^	ΔPANSS negative	53	−71.6	0.5948
	ΔPANSS positive	53	−74.5	0.6824
	ΔPANSS disorganization	53	−35.3	0.8493
	ΔPANSS depression/anxiety	52	222.7	0.1173
	^a^ΔPANSS excitement	53	473.5	0.0034
^b^ΔCDSS		49	717.2	0.0006

Delta was calculated subtracting the measures of follow-up minus the baseline

^a^Significant *p*-values without Bonferroni correction

^b^Significant *p*-values with Bonferroni correction

Within FEP subtypes, depressive symptoms (CDSS) were positively associated with PRS in both FEP subtypes when analysed separately at baseline (B_schizophrenia_ = 1746.1; p_schizophrenia_ = 0.002; B_schizophreniform = _2660.0; p_schizophreniform = _0.036), while PANSS excitement and −GAF was associated only in the schizophreniform subgroup (*B* = 1393.3; *p* = 9 × 10^-5^ and *B* = −1449.1; *p* = 1.3 × 10^-4^, respectively) (Table 4, Supplementary Figs S5 and S6).

**Table 4:** Association between the statistically significant clinical variables with PRS splitting the FEP patients into FEP subtypes according to the follow-up diagnosis

Subtype	Clinical variable	Timepoint	*N*	B	*p*-value
Schizophreniform	^a^−GAF	Baseline	9	−1449.1	0.00013
	^b^CDSS	Baseline	9	2660.0	0.03594
	CDSS	Follow-up	10	−2323.7	0.20033
	^a^PANSS excitement	Baseline	11	1393.3	0.00009
	PANSS depression/anxiety	Follow-up	10	−622.3	0.15126
	^b^ΔPANSS excitement	Baseline - follow-up	11	877.8	0.02002
	ΔCDSS	Baseline - follow-up	9	497.4	0.30954
Schizophrenia only	−GAF	Baseline	27	300.3	0.21538
	^b^CDSS	Baseline	29	1746.1	0.00217
	^a^CDSS	Follow-up	27	−3739.3	0.00009
	PANSS excitement	Baseline	29	206.5	0.39645
	^a^PANSS depression/anxiety	Follow-up	29	−1286.2	0.00002
	ΔPANSS excitement	Baseline - follow-up	29	324.9	0.19858
	^a^ΔCDSS	Baseline - follow-up	27	1358.7	0.00002

^a^Significant *p*-values with Bonferroni correction

^b^Significant *p*-values without Bonferroni correction

Given the different pattern of association between depressive symptoms (CDSS) and PRS at the baseline (positive association) and after treatment (negative association), we generated a trajectory plot to visualize each individual symptom in both time points (Figure S9). We can note that those individuals with high PRS tend to show a decrease in their depressive symptoms after risperidone treatment, while those with low PRS tend to maintain or increase their level of depressive symptoms.

## Discussion

In this study, we demonstrate for the first time that the SCZ PRS is associated with different clinical symptoms during the pre-treatment stage of FEP. Although recently Sengupta et al.^17^ reported positive associations between PRS and clinical variables and FEP, it is important to note that our methods were different in many aspects; we included (1) only non-affective FEP, (2) only antipsychotic naive FEP, (3) applied a standardized treatment (risperidone) and (4) calculated the PRS using more than 21 K SNPs (compared with 84 used by them). Specifically, in pre-treatment FEP patients, we identified a positive correlation of PRS with depressive symptoms (CDSS total), excitement symptoms (PANSS-excitement factor) and with Global Assessment of Functioning (−GAF). After standardized treatment for 9 weeks with risperidone, we observed no positive association for these or other clinical measurements, but a negative correlation with PRS emerges for both CDSS and PANSS depressive/anxiety factor. Concordant with this, Sengupta et al.^17^ observed similar results for CDSS, although not reaching statistical significance, probably because of the lower power of including only 84 SNPs in the genetic risk score. These results suggest the potential aetiological importance of depression (and anxiety) in SCZ. In sensitivity analyses, we found that observed baseline positive correlation with PANSS excitement and –GAF was driven by the subgroup composed by individuals with schizophreniform or brief psychosis disorder diagnoses (Table 4), while the positive correlation of SCZ PRS with depressive symptoms was present irrespective of diagnostic group.

Looking at treatment response, we found that pre-treatment baseline to post-treatment follow-up changes for PANSS excitement (ΔPANSS excitement**)** and CDSS (ΔCDSS) were also positively correlated with SCZ PRS (Table 3), suggesting that patients with a higher PRS tend to show more improvement in symptoms after treatment (Figure S9) and that those with lower PRS have increased depressive symptoms. It is well known that some patients may have an increase in depressive symptoms once positive symptoms remit, being recognised as a clinical disorder (ICD F20.4 = post-SCZ depression). However, no study, to our knowledge has yet evaluated the relation between PRS and post-SCZ depression. Lastly, it is important to note that although we detected differences in these two symptom dimensions, we do not observe an association between total PANSS improvement (ΔPANSS total) and PRS SCZ. Taken together, these results suggest that FEP patients who present with higher depressive and excitement symptoms and/or who show reduction in these dimensions after treatment with risperidone have a significantly higher genetic risk for SCZ (as estimated by PRS).

It is important to note that all previous studies of PRS and symptoms in SCZ used different study designs and, moreover, their samples were composed of patients under antipsychotic treatment. Vassos et al.^15^ suggested that the different subgroups in theirFEP sample (specifically non-affective versus affective psychosis) have different PRS, whereas our sample included only non-affective FEP. Additionally, we did not observe overall PRS differences between our FEP subgroups. In addition, a recent study, within a large population cohort of adolescents, found an association between PRS and negative symptoms but not with depressive symptoms^16^.

One strength of our study is that all patients were antipsychotic-naïve at the baseline and received the same treatment for approximately the same time. Our study also has several limitations, primary amongst which is that our FEP sample size is small (*N* = 60). The treatment used, risperidone, has been shown to be beneficial as an augmentation therapy in MDD patients who have a high-risk for suicide^23^ and in patients who were treatment-refractory for MDD^24^. However, it is a unique longitudinal sample of antipsychotic naïve FEP individuals. We will increase this sample in the future but at the moment it represents a (near) unique resource. Despite these limitations, this is the first study to explore PRS before standardized treatment initiation in the FEP as well as the change in symptoms after a sufficient time period in which to observe response to treatment.

Our results suggest that drugs and other treatments may confound our understanding of the influence of PRS on symptomatology due to their effects on specific symptoms. We expect that future studies will explore additional clinical dimensions, taking into account the response to different antipsychotics, while increasing the sample size of treatment naïve patients analysed to have more statistical power. In conclusion, we have shown that that excitement and depressive symptoms are positively associated to SCZ-PRS during FEP pre-treatment but not after risperidone treatment and that increased SCZ-PRS is associated with the risperidone induced improvement of both depressive and excitement symptoms.

## Disclaimer

The views expressed are those of the author(s) and not necessarily those of the UK NHS, the UK NIHR or the UK Department of Health. Results reflect the author’s view and the Agency is not responsible for any use that may be made of the information it contains.

## Electronic supplementary material

Supplemental Material
