# Supplementary material for: Polygenic risk score analyses of symptoms and treatment response in an antipsychotic-naive first episode of psychosis cohort
Source: Transl Psychiatry. 2018 Aug 31;8:174. doi: 10.1038/s41398-018-0230-7 (PMC6119191; doi:10.1038/s41398-018-0230-7)

**Supplementary Table S1.** Five-factor model validated by Higuchi et al. 2014.

| Five-Factor Model | ITEMS | Question |
| --- | --- | --- |
| NEGATIVE | Poor rapport | N3 |
|  | Lack of spontaneity | N6 |
|  | Emotional withdrawal | N2 |
|  | Passive/apathetic social withdrawal | N4 |
|  | Blunted affect | N1 |
|  | Motor retardation | G7 |
| DISORGANIZATION / COGNITION | Conceptual disorganization | P2 |
|  | Poor attention | G11 |
|  | Disorientation | G10 |
|  | Disturbance of volition | G13 |
|  | Difficulty in abstract thinking | N5 |
|  | Stereotyped thinking | N7 |
|  | Mannerisms/posturing | G5 |
| EXCITEMENT | Uncooperativeness | G8 |
|  | Poor impulse control | G14 |
|  | Hostility | P7 |
|  | Excitement | P4 |
| POSITIVE | Delusions | P1 |
|  | Unusual thought content | G9 |
|  | Hallucinatory behavior | P3 |
|  | Suspiciousness/persecution | P6 |
|  | Grandiosity | P5 |
| DEPRESSION / ANXIETY | Anxiety | G2 |
|  | Guilt feelings | G3 |
|  | Depression | G6 |
|  | Tension | G4 |

**Supplemental Table S2.** Demographics association with the best p-threshold PRS. The demographics were available only for patients.

|  | N | B | p-value |
| --- | --- | --- | --- |
| Familial Income (US$) | 48 | 1050797.9 | 0.1027 |
| Other Drugs | 41 | 426.4 | 0.3833 |
| Cannabis | 25 | 616 | 0.2417 |
| BMI | 34 | 8929.5 | 0.0569 |
| Familial History | 46 | -274.4 | 0.524 |

**Supplemental Table S3.** Demographics association with the clinical variables. We used t.test() function for Other drugs and Cannabis and cor.test() for Familial income and BMI.

| **Clinical variables** | **Other drugs** | | | **Cannabis** | | | **Familial income** | | | **BMI** | | |
| --- | --- | --- | --- | --- | --- | --- | --- | --- | --- | --- | --- | --- |
|  | **t** | **df** | **p-value** | **t** | **df** | **p-value** | **t** | **df** | **p-value** | **t** | **df** | **p-value** |
| **PANSStot_1** | -0.27 | 23 | 0.791 | -0.28 | 30 | 0.779 | -0.46 | 46 | 0.650 | -0.30 | 21 | 0.769 |
| **PANSStot_2** | -0.39 | 20 | 0.703 | 0.51 | 29 | 0.614 | 1.81 | 44 | 0.077 | 0.34 | 20 | 0.735 |
| **CGI_total_1** | 0.56 | 24 | 0.582 | 0.85 | 27 | 0.403 | 0.40 | 45 | 0.690 | 0.26 | 20 | 0.800 |
| **GAF_1** | 0.12 | 23 | 0.905 | 0.11 | 24 | 0.915 | -0.04 | 43 | 0.972 | -0.88 | 20 | 0.389 |
| **CGI_total_2** | 0.91 | 19 | 0.374 | 1.25 | 27 | 0.223 | -0.76 | 42 | 0.452 | 0.53 | 20 | 0.600 |
| **GAF_2** | -0.19 | 18 | 0.855 | -0.60 | 28 | 0.552 | 0.03 | 43 | 0.973 | -0.21 | 21 | 0.835 |
| **calgarytot_1** | 0.60 | 24 | 0.555 | 0.86 | 18 | 0.399 | 0.30 | 44 | 0.767 | 1.47 | 21 | 0.155 |
| **calgarytot_2** | -0.43 | 25 | 0.669 | -0.23 | 27 | 0.823 | 0.25 | 44 | 0.807 | 1.18 | 20 | 0.251 |
| **PANSS_Neg_1** | -0.12 | 24 | 0.903 | -0.03 | 24 | 0.976 | -0.41 | 43 | 0.688 | -1.08 | 19 | 0.295 |
| **PANSS_Neg_2** | 0.22 | 19 | 0.825 | 0.88 | 27 | 0.385 | 1.63 | 42 | 0.112 | -0.62 | 19 | 0.542 |
| **PANSS_Pos_1** | -1.05 | 21 | 0.307 | -1.68 | 28 | 0.105 | 0.11 | 43 | 0.912 | 0.35 | 19 | 0.732 |
| **PANSS_Pos_2** | -0.54 | 24 | 0.596 | -0.22 | 20 | 0.829 | 0.11 | 42 | 0.912 | -0.19 | 19 | 0.854 |
| **5F-PANSS_neg1** | -0.37 | 25 | 0.714 | -0.19 | 28 | 0.849 | -0.30 | 46 | 0.764 | 0.02 | 21 | 0.981 |
| **5F-PANSS_dis1** | 0.12 | 24 | 0.904 | 0.21 | 27 | 0.834 | -0.24 | 46 | 0.809 | -0.99 | 21 | 0.332 |
| **5F-PANSS_exc1** | -1.15 | 23 | 0.261 | -1.57 | 29 | 0.128 | -0.13 | 46 | 0.899 | 0.56 | 21 | 0.579 |
| **5F-PANSS_pos1** | 0.23 | 21 | 0.822 | -0.48 | 30 | 0.632 | -0.09 | 46 | 0.933 | 0.34 | 21 | 0.734 |
| **5F-PANSS_dep1** | -0.63 | 23 | 0.538 | -0.31 | 30 | 0.756 | -0.76 | 46 | 0.453 | -0.22 | 21 | 0.826 |
| **5F-PANSS_neg2** | -0.05 | 19 | 0.963 | 0.56 | 29 | 0.582 | 2.50 | 46 | 0.016 | 0.17 | 21 | 0.866 |
| **5F-PANSS_dis2** | -0.73 | 19 | 0.475 | 0.19 | 29 | 0.852 | 2.05 | 46 | 0.046 | -0.07 | 21 | 0.948 |
| **5F-PANSS_exc2** | -0.82 | 25 | 0.420 | -0.34 | 27 | 0.734 | 0.36 | 46 | 0.720 | 0.47 | 21 | 0.640 |
| **5F-PANSS_pos2** | 0.71 | 27 | 0.482 | 0.96 | 21 | 0.348 | -0.24 | 46 | 0.812 | 0.22 | 21 | 0.830 |
| **5F-PANSS_dep2** | -0.42 | 21 | 0.681 | -0.01 | 30 | 0.995 | 1.28 | 45 | 0.208 | 0.15 | 21 | 0.879 |

*1 = baseline; *2 = follow-up; *tot* = total score ; 5F-PANSS* = Five- factor PANSS (Higuchi et al.,); *Neg* = negative symptoms; *pos* = positive symptoms; *exc* = excitement symptoms; *dep* = depressive symptoms; *dis* = disorganization / cognition symptoms

**Supplementary Table S4.** PRSice results, explained variability and number of SNPs for each threshold.

| Threshold | p.out (Naegelkerke’s) | r2.out | Nsnps |
| --- | --- | --- | --- |
| 0.001 | 0.0032 | 0.10 | 5533 |
| 0.01 | 0.0003 | 0.16 | 20223 |
| 0.0112 | 0.0001 | 0.19 | 21622 |
| 0.05 | 0.0004 | 0.15 | 53790 |
| 0.1 | 0.0004 | 0.15 | 82978 |
| 0.2 | 0.0007 | 0.14 | 126514 |
| 0.3 | 0.0011 | 0.13 | 160713 |
| 0.4 | 0.0018 | 0.12 | 188576 |
| 0.5 | 0.002 | 0.12 | 211371 |

**Supplementary Table S5**. Comparison between Caucasian only analysis and all individuals

|  | estimate_caucasians | pvals_caucasians | N_caucasians | Estimate_all | pvals_all | N_all |
| --- | --- | --- | --- | --- | --- | --- |
| GAF_1 | -859.90 | 2x10^-06^** | 32 | -436.13 | 0.0029* | 48 |
| GAF_2 | 54.55 | 0.7174 | 33 | 132.82 | 0.2281 | 53 |
| calgarytot_1 | 674.45 | 0.1579 | 35 | 1042.25 | 0.0039* | 51 |
| calgarytot_2 | -1354.06 | 0.039* | 34 | -1800.15 | 0.0004** | 53 |
| panss_exc1 | 639.27 | 0.0018* | 35 | 566.67 | 0.0003** | 53 |
| panss_depa2 | -206.09 | 0.3767 | 35 | -575.02 | 0.0013* | 55 |

**Supplementary Figure S1.** Ten Principal components for cases and controls separately.


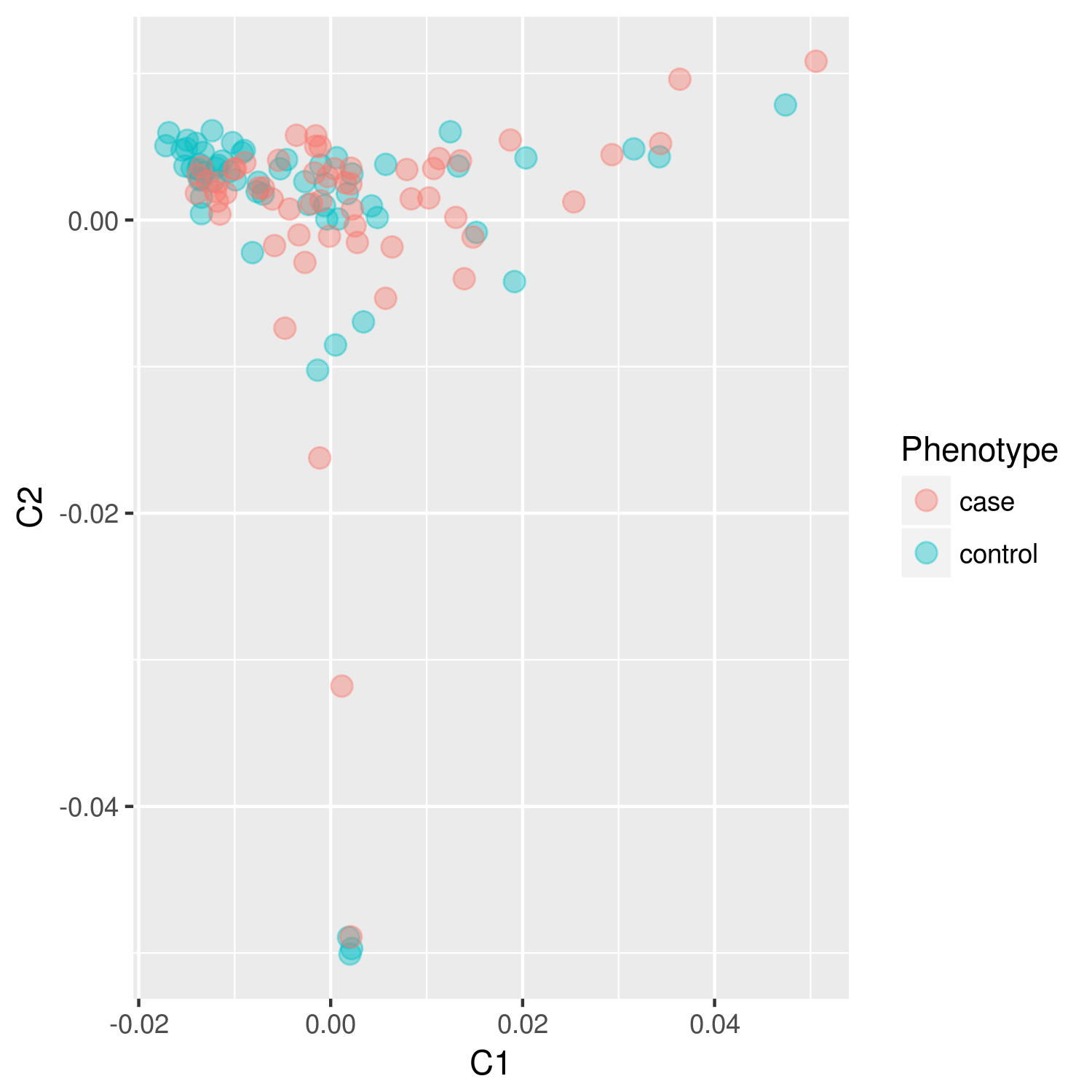

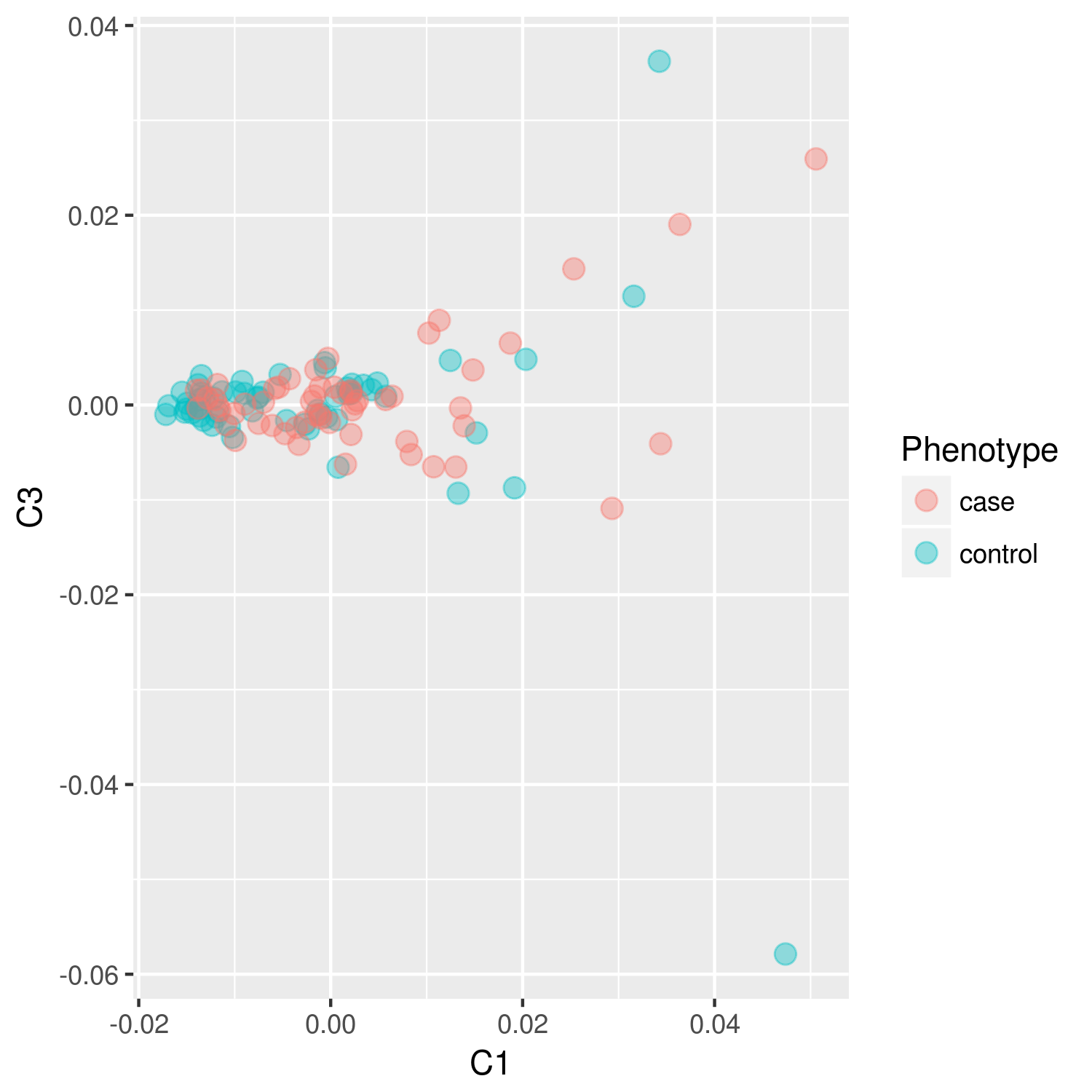

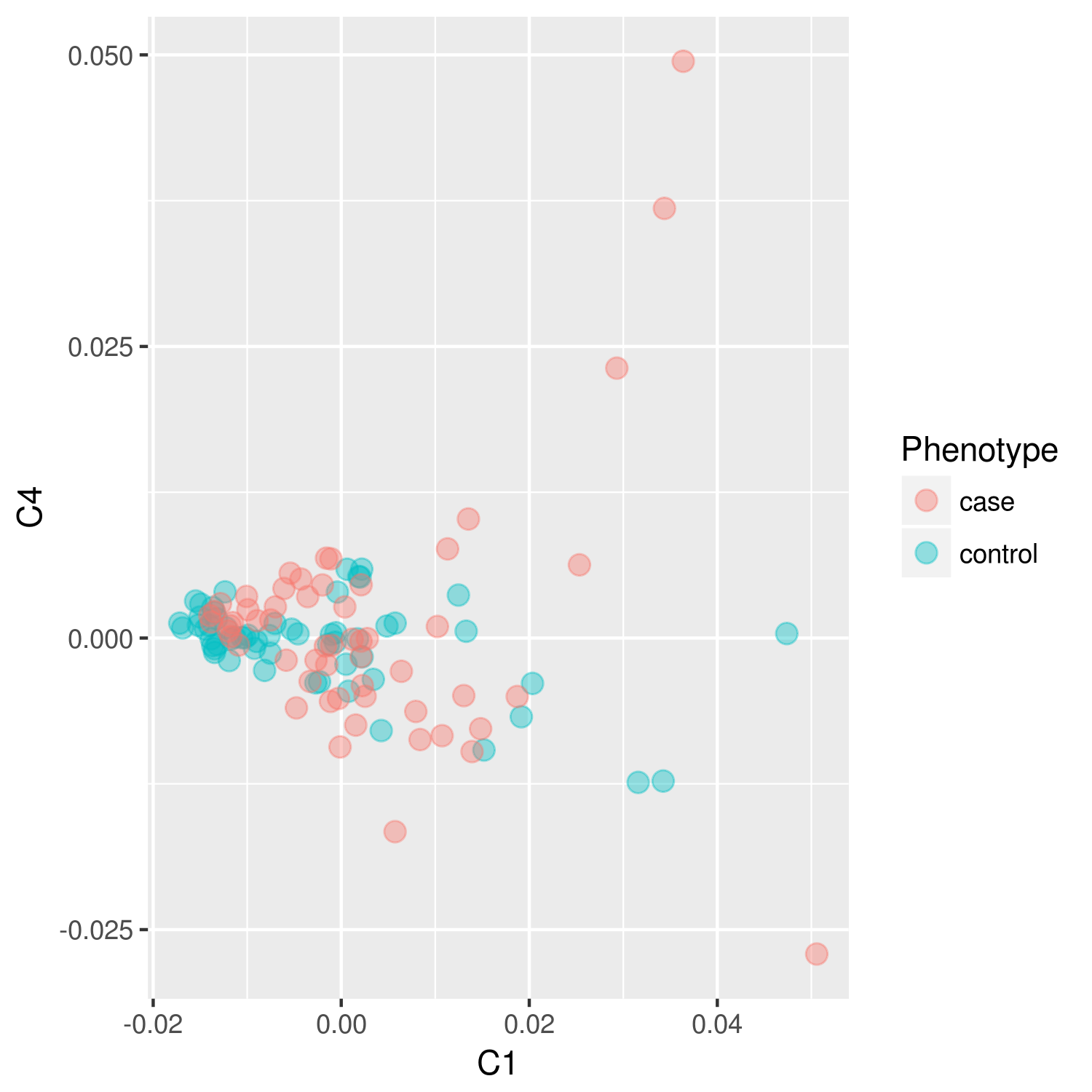

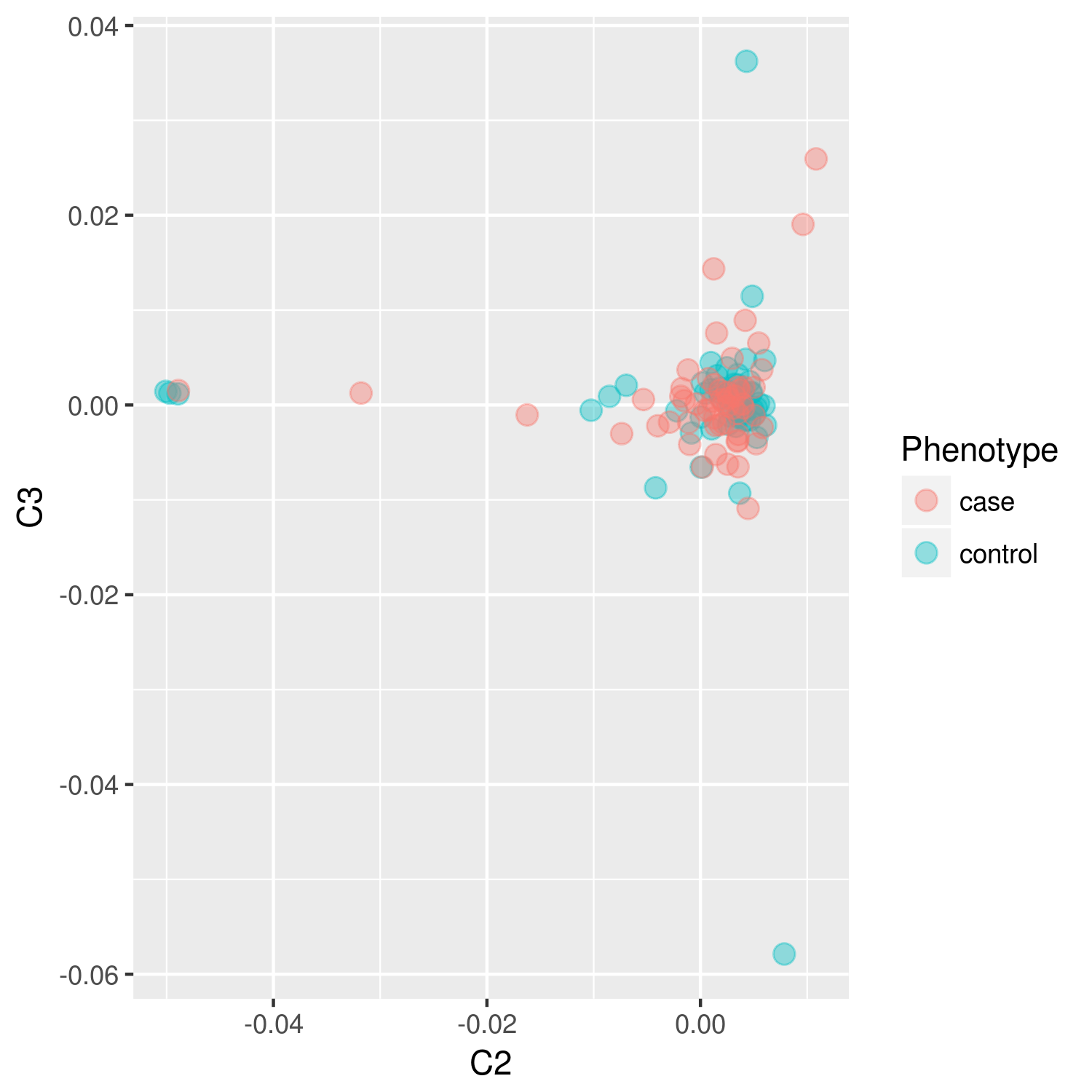

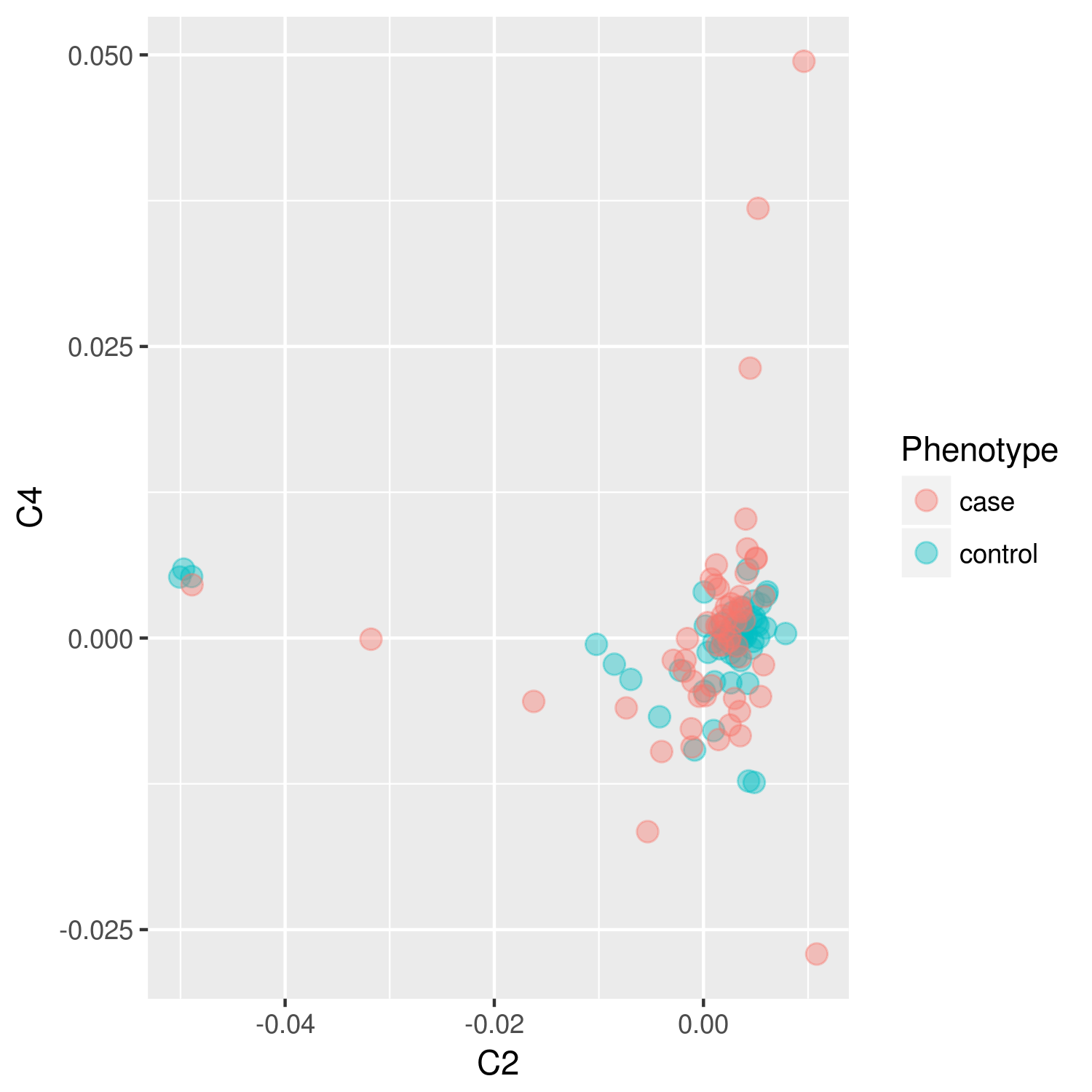

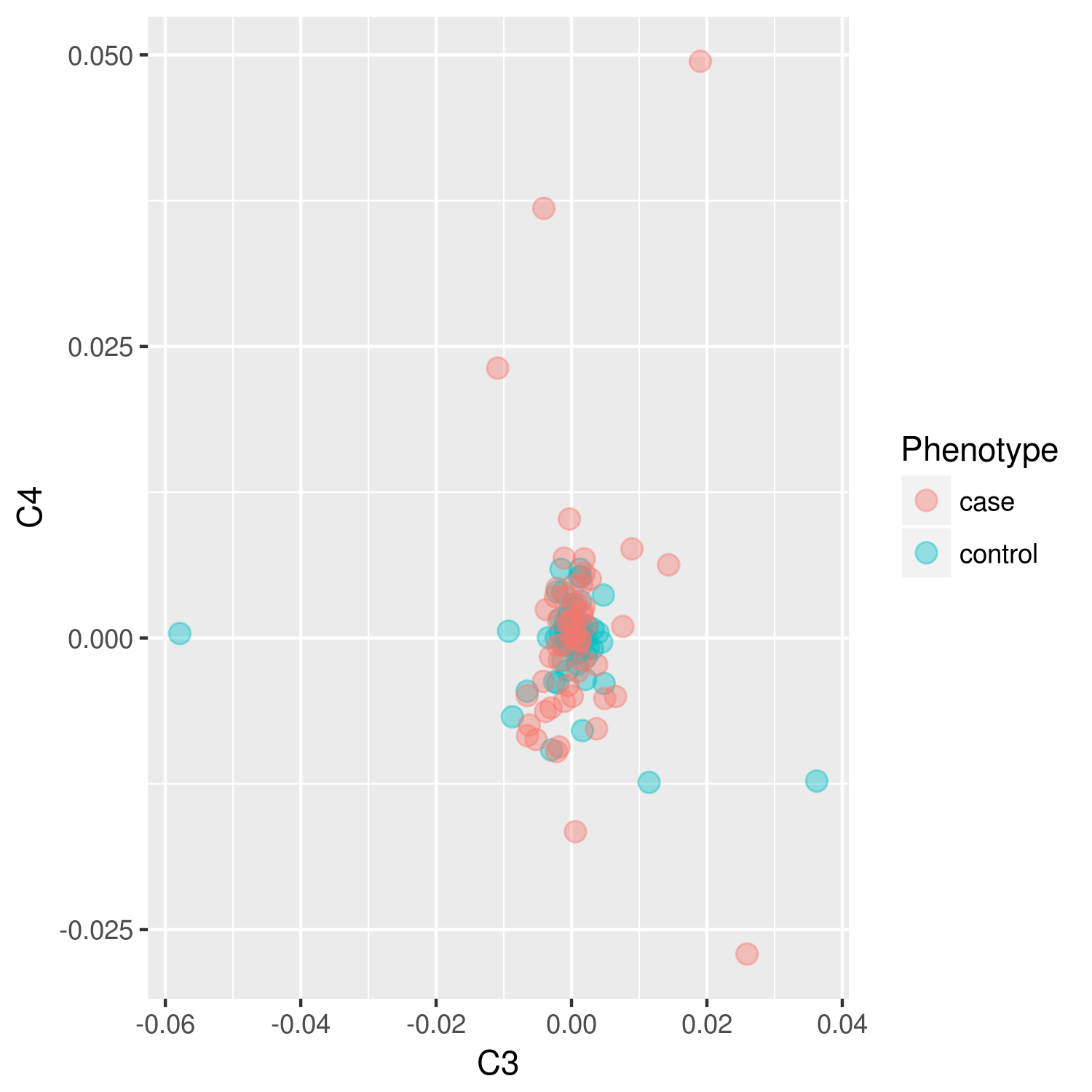

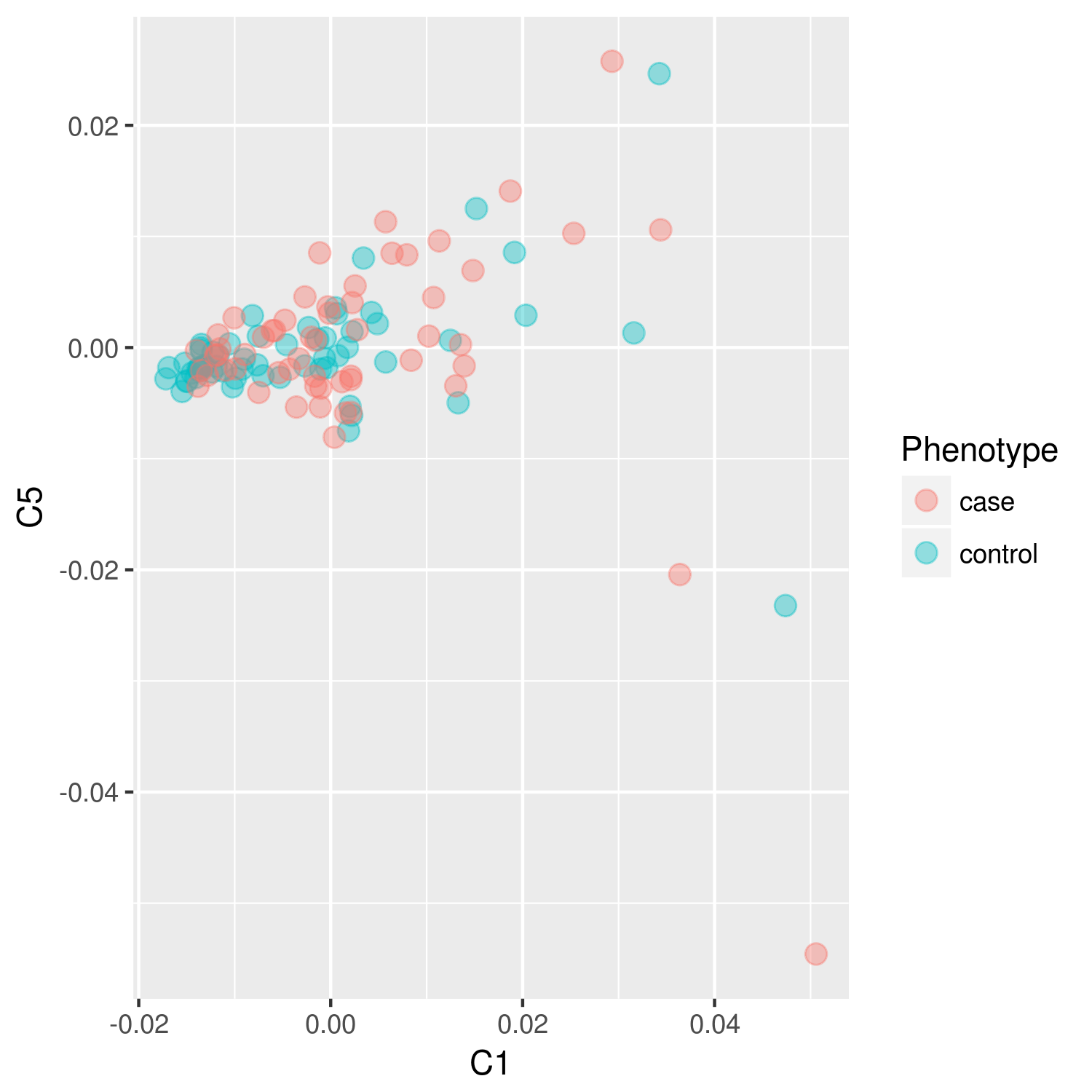

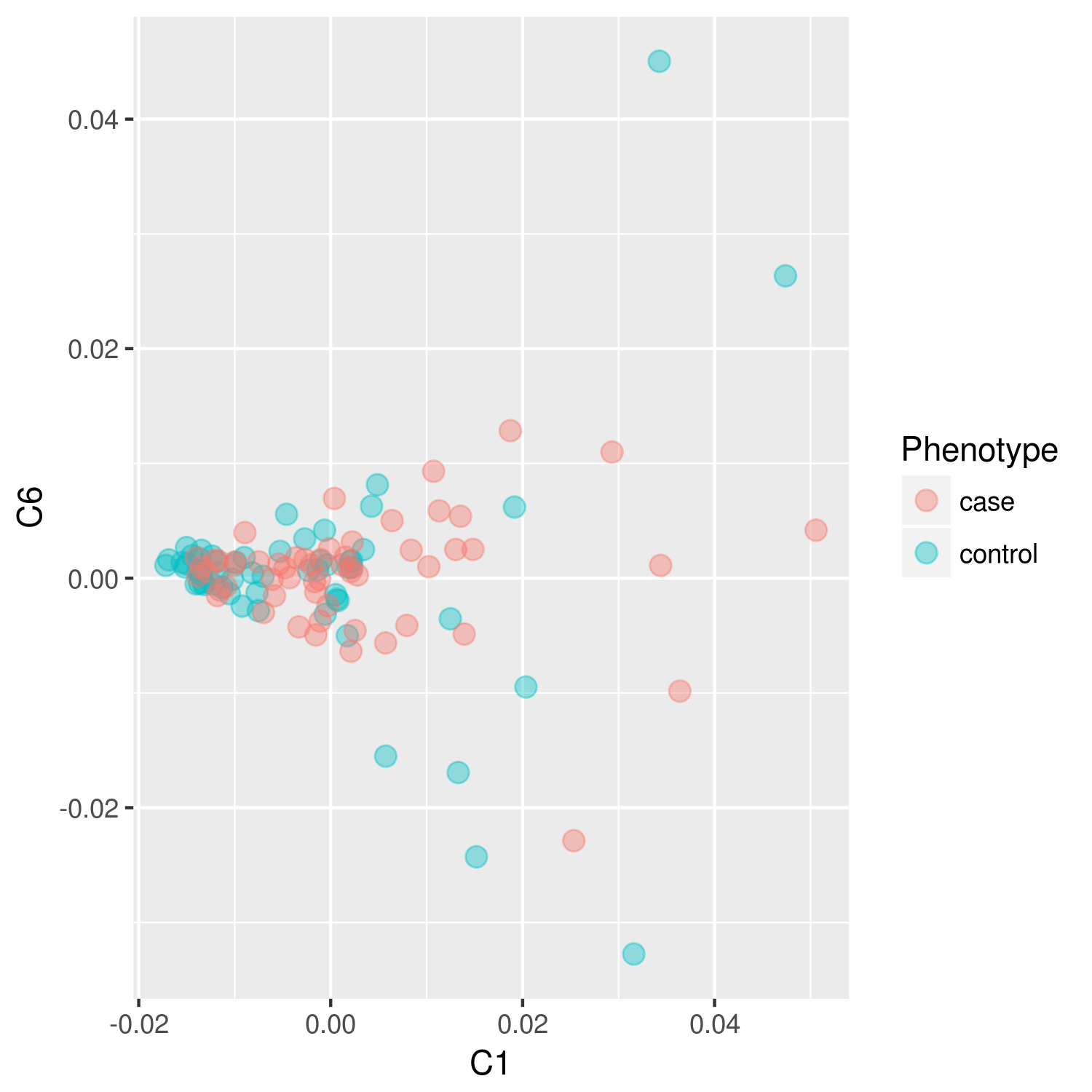

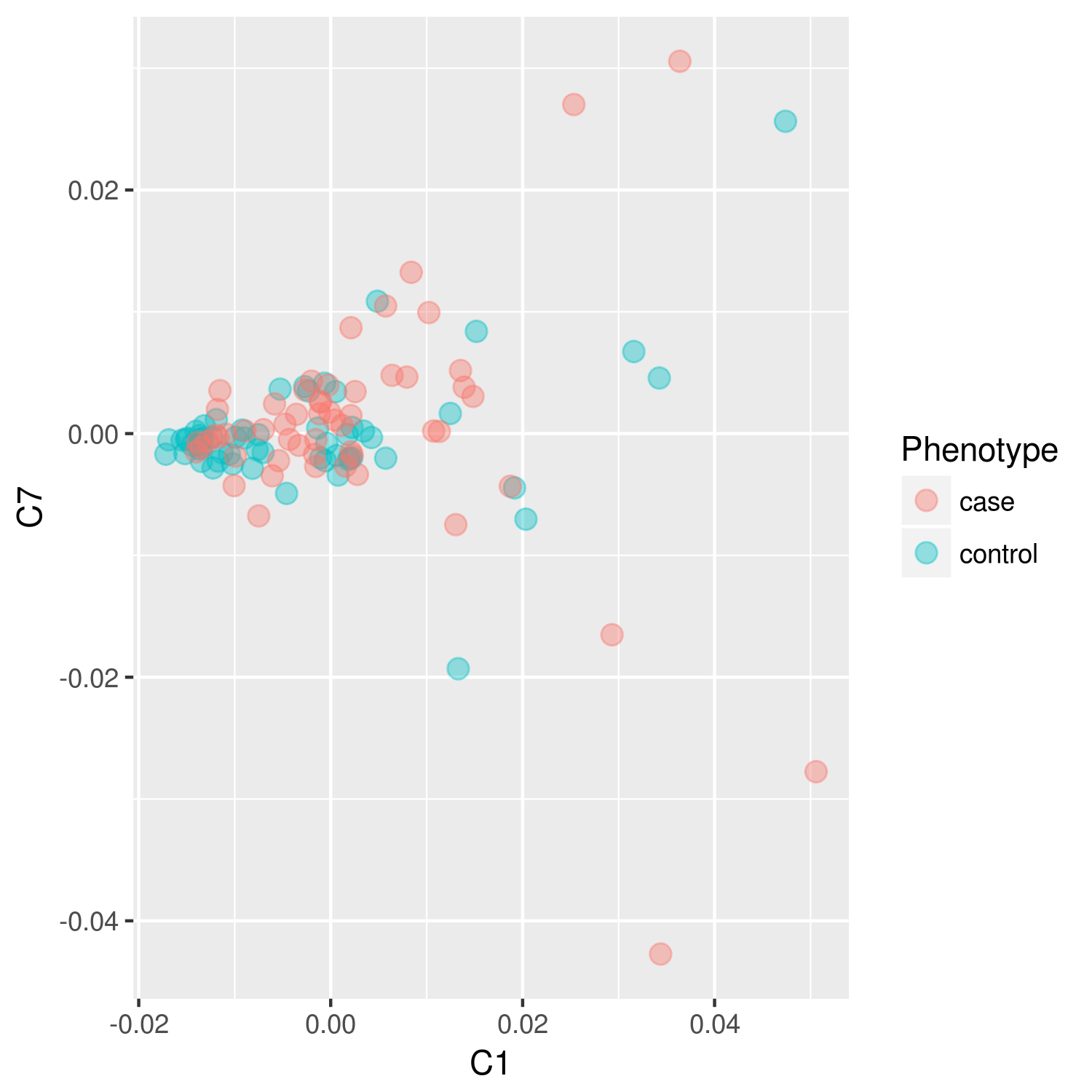

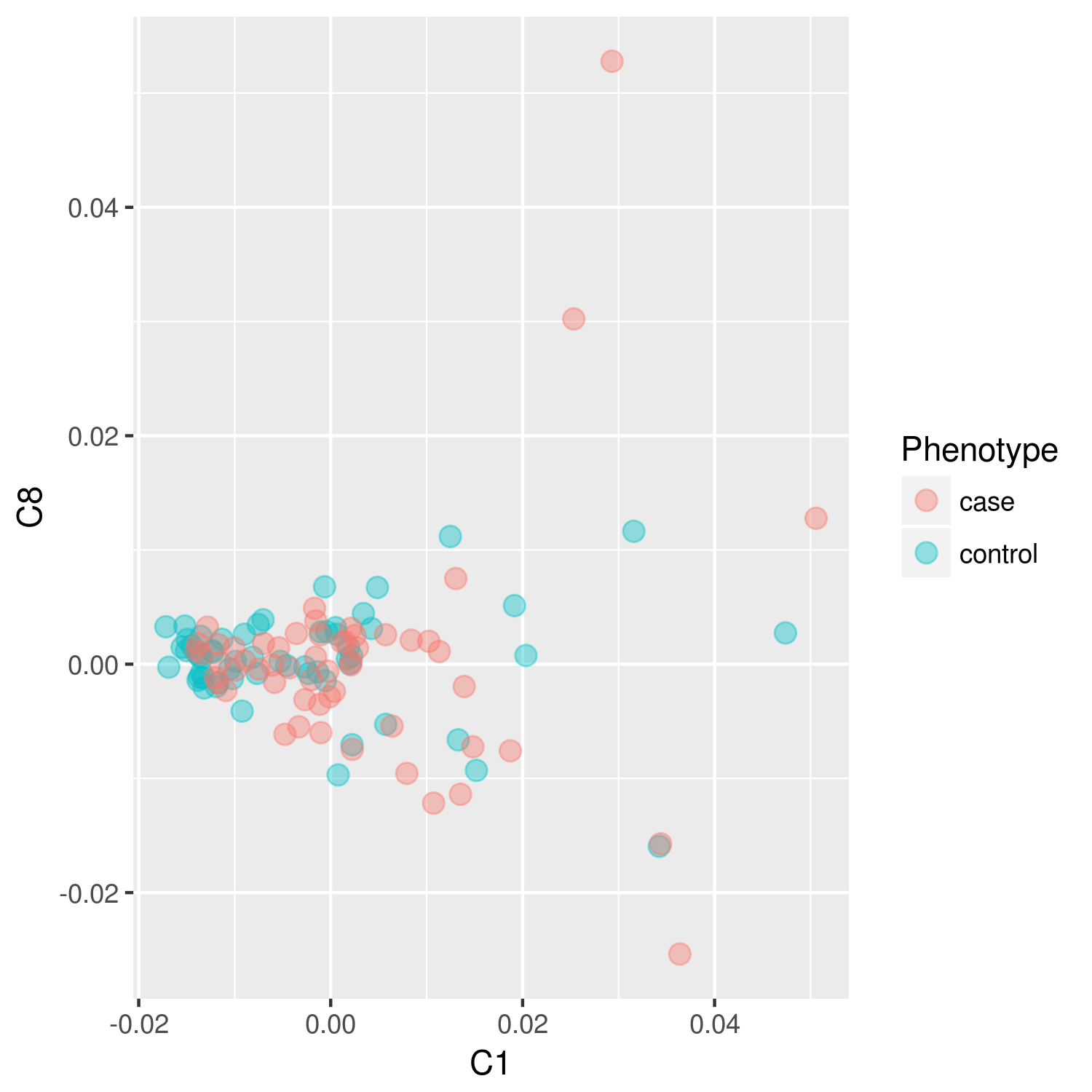

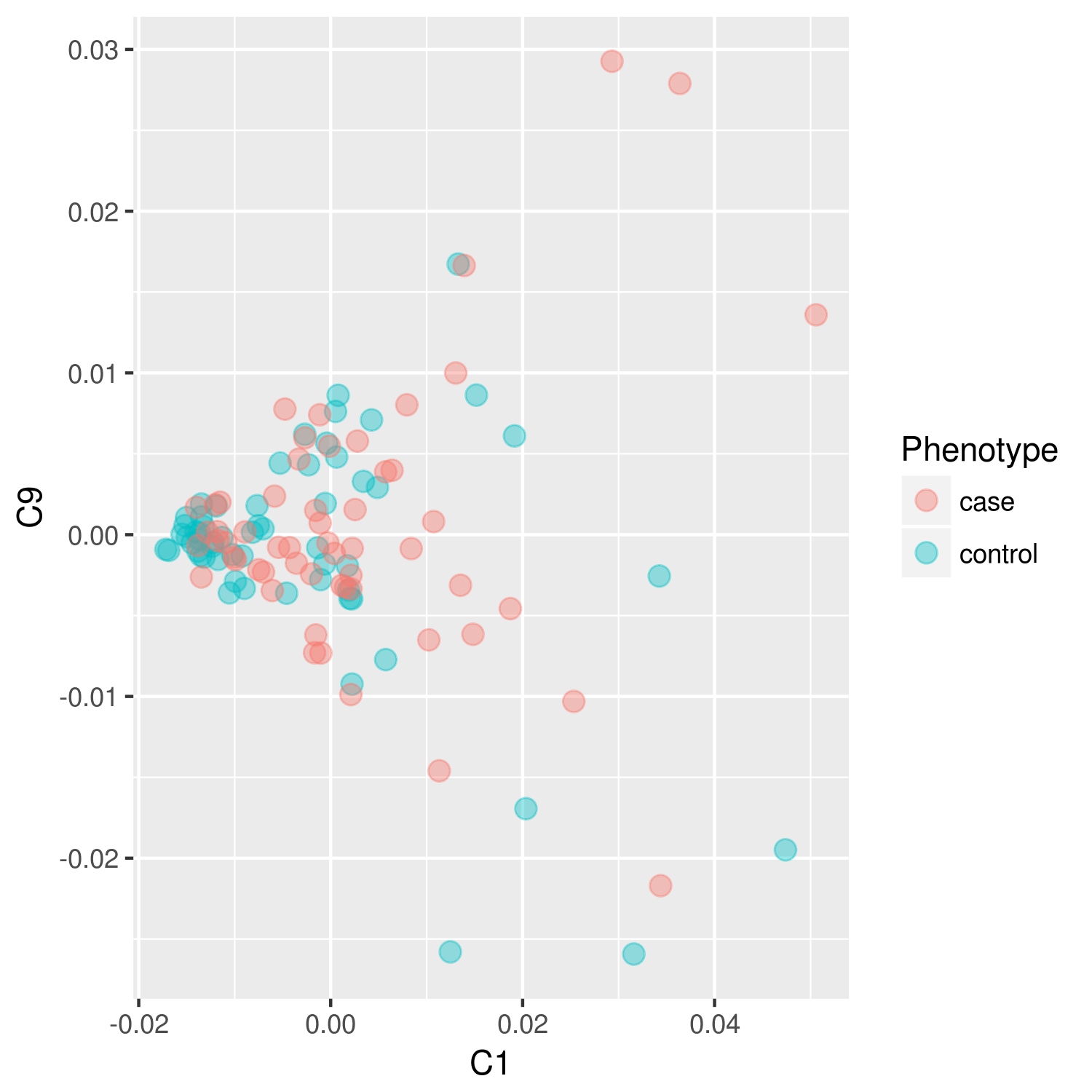

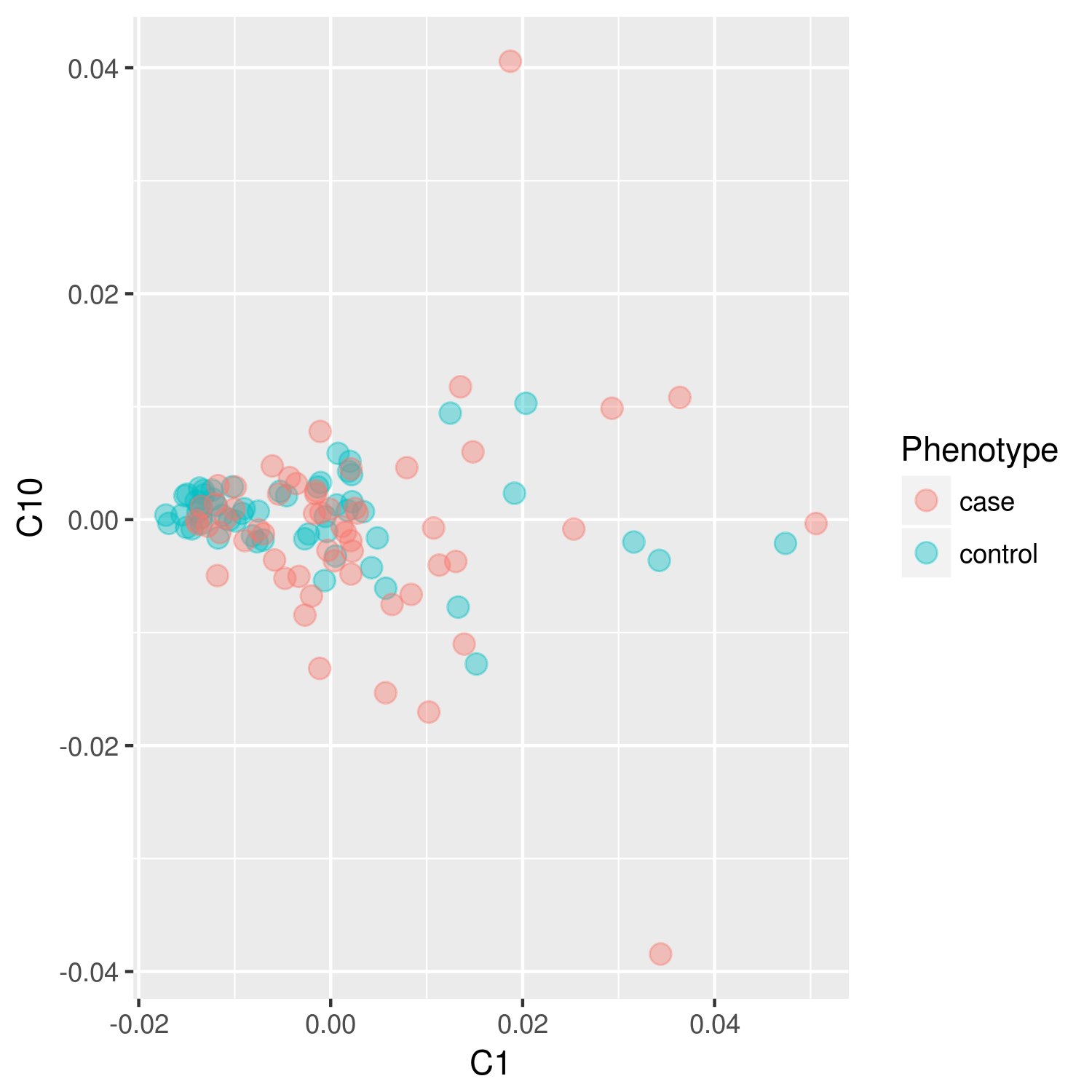


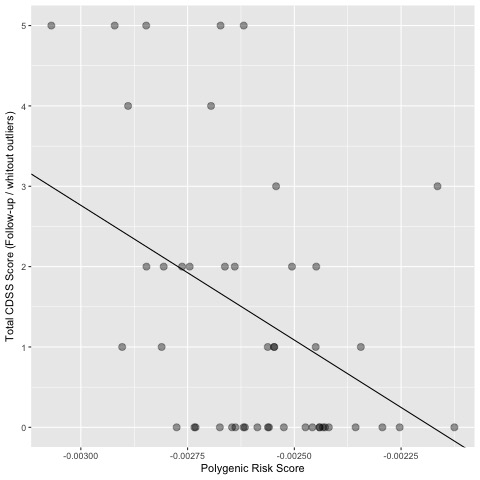

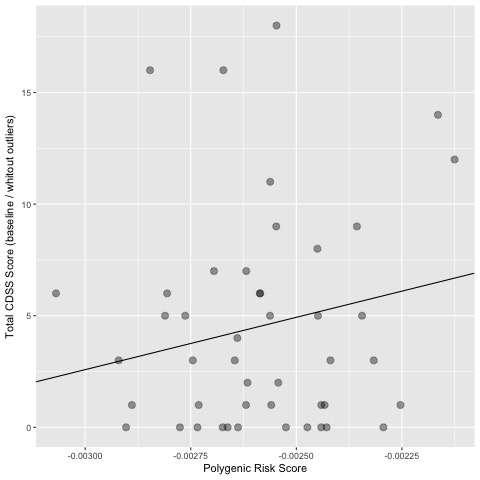
**Supplementary Figure S2.** Polygenic Risk Scores and CDSS measurements plots for both time points and Delta CDSS excluding outlier individuals for CDSS (6 patients).


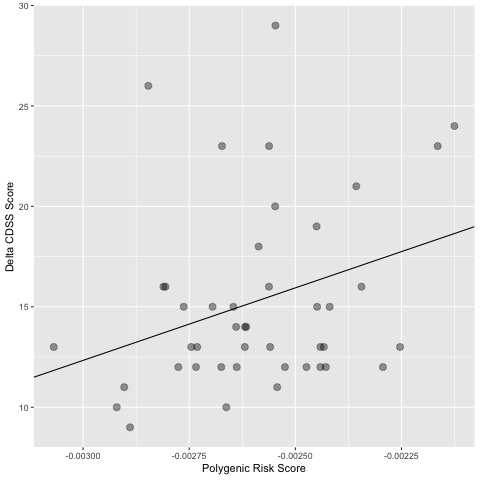


**Supplementary Figure S3.** Polygenic Risk Scores and baseline PANSS excitement factor measurement plot.


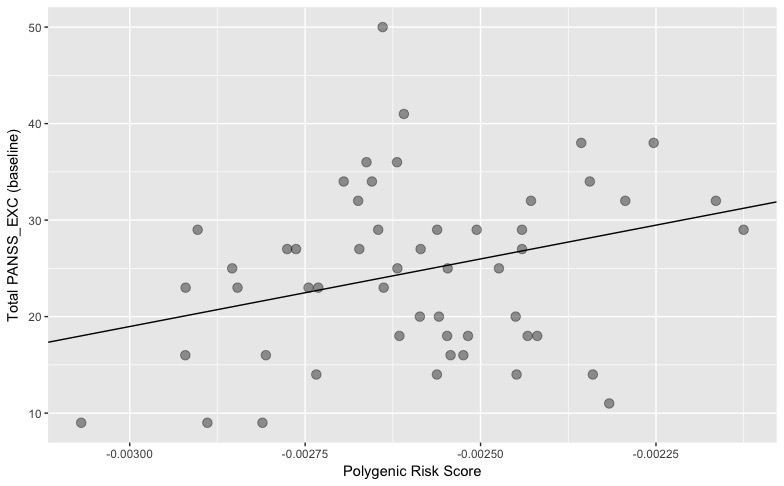


**Supplementary Figure S4.** Polygenic Risk Scores and Delta PANSS total plot.


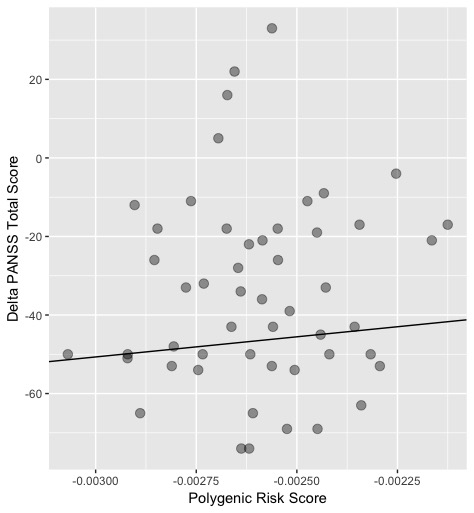


**Supplementary Figure S5.** Polygenic Risk Score and statistical significant measures, confirmatory analysis selecting only **schizophreniform** patients.


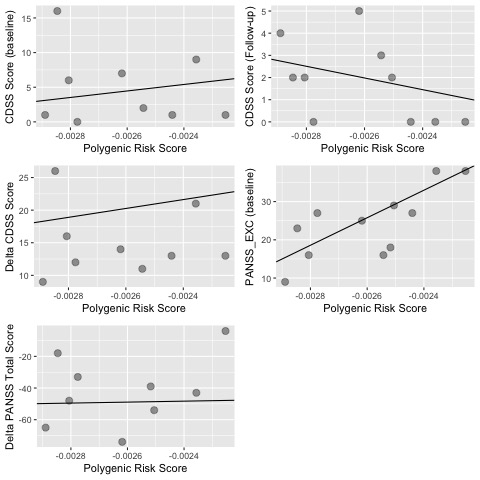


**Supplementary Figure S6.** Polygenic Risk Score and statistical significant measures, confirmatory analysis selecting only **schizophrenia** patients.


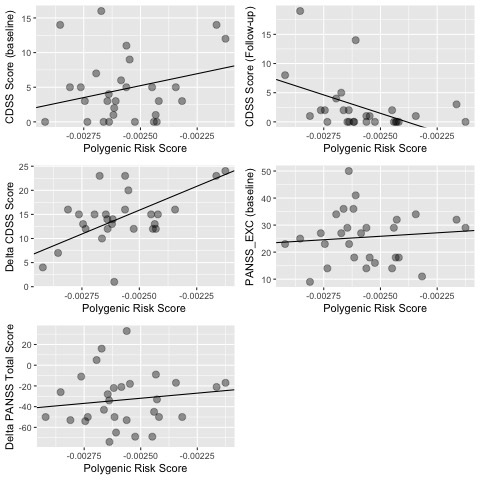


**Supplementary Figure S7.** Polygenic Risk Scores distribution for all cases, Caucasian cases (self-declared) and all controls.


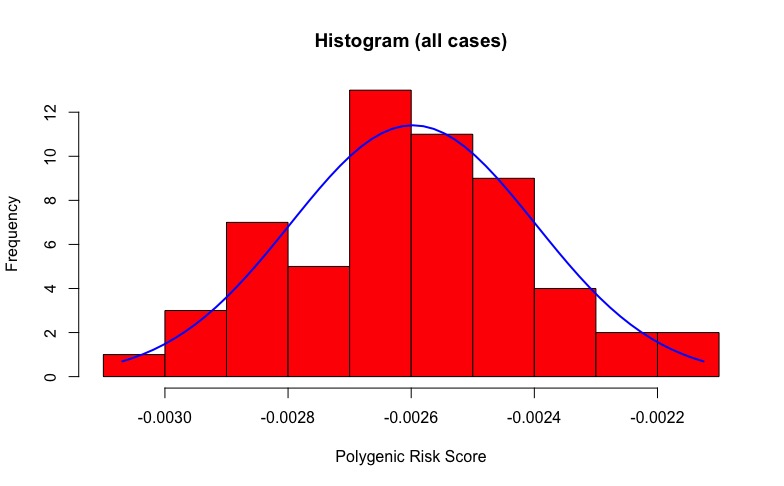


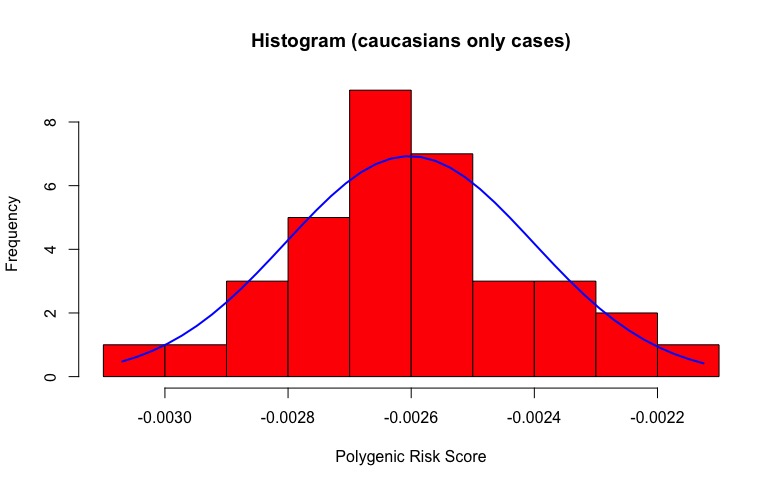

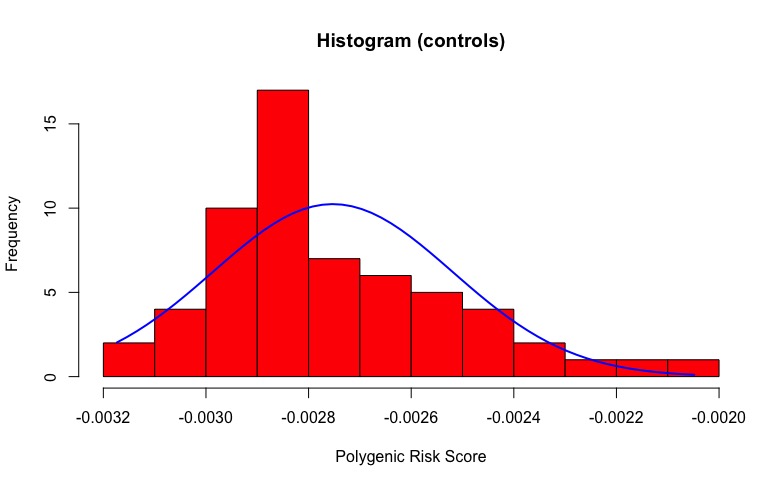


**Supplementary Figure S8.** Heatmap correlation plot for all clinical measurements for both time points.


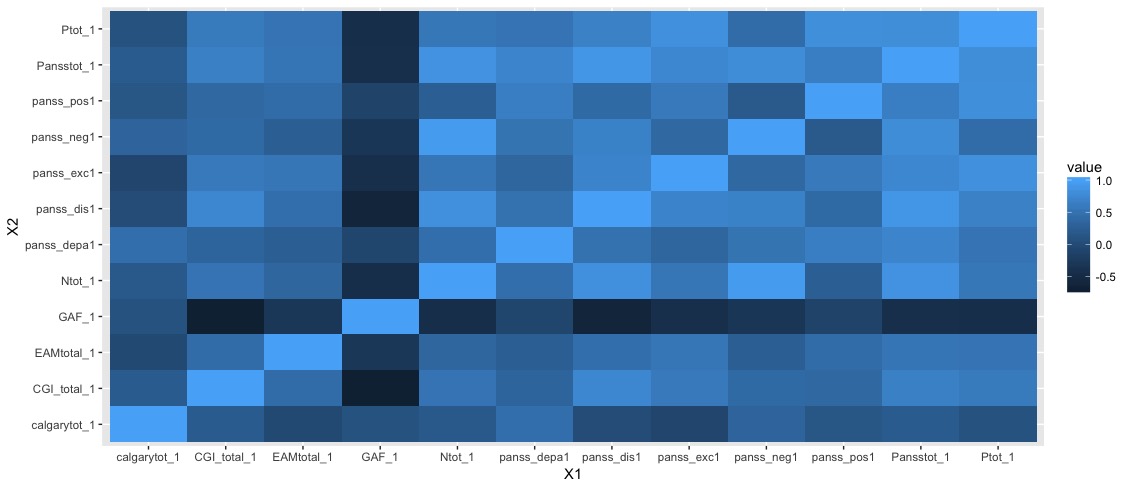

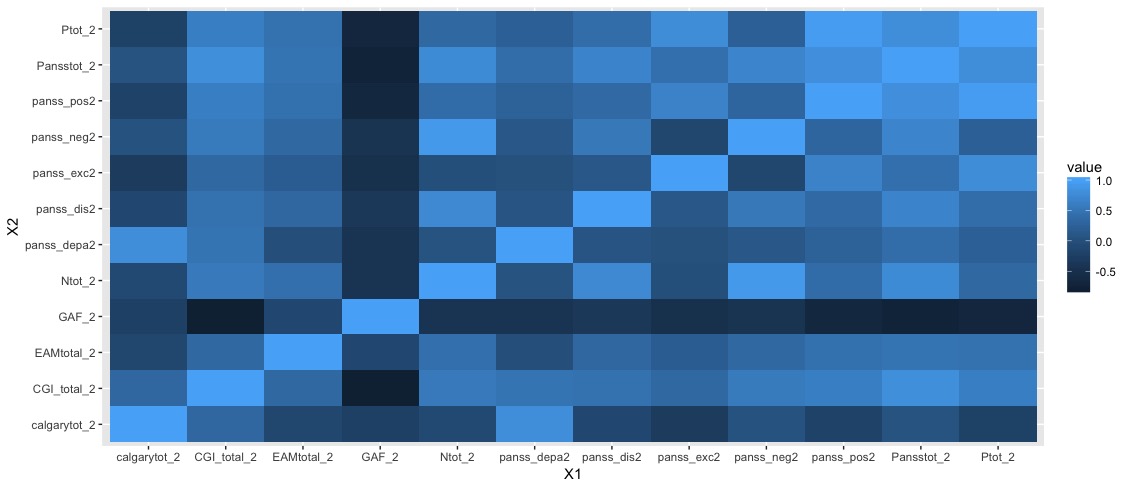


**Supplementary Figure S9.** CDSS trajectory of each individual from baseline to follow-up and their PRS.


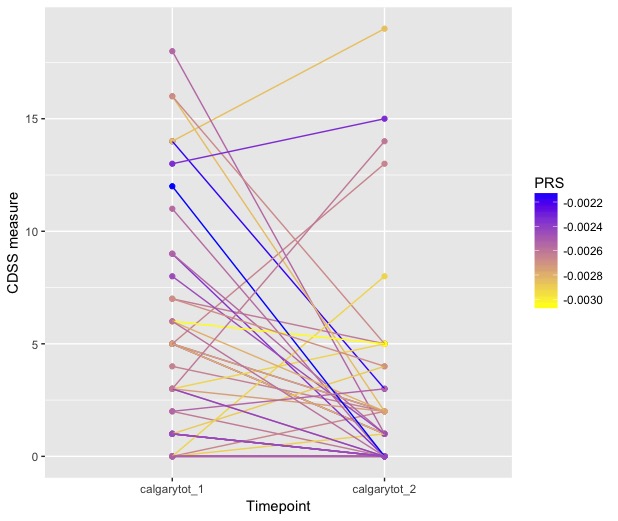


**Supplementary Figure S10**. Odds ratios (ORs) of psychosis at different levels of polygenic risk score (PRS). For more information on how the OR were calculated please see Vassos et al.,2017.


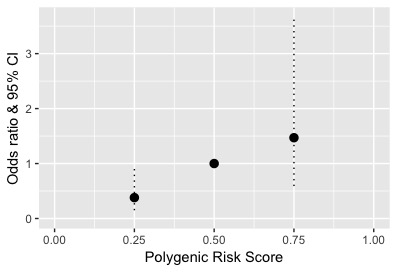

Supplement: Supplementary file 1 — Supplemental Material [file 41398_2018_230_MOESM1_ESM.docx]
